# Changes of the human skin microbiota upon chronic exposure to polycyclic aromatic hydrocarbon pollutants

**DOI:** 10.1186/s40168-020-00874-1

**Published:** 2020-06-26

**Authors:** Marcus H. Y. Leung, Xinzhao Tong, Philippe Bastien, Florent Guinot, Arthur Tenenhaus, Brice M. R. Appenzeller, Richard J. Betts, Sakina Mezzache, Jing Li, Nasrine Bourokba, Lionel Breton, Cécile Clavaud, Patrick K. H. Lee

**Affiliations:** 1 School of Energy and Environment, City University of Hong Kong, Hong Kong SAR, China; 2 L’Oréal Research and Innovation, Aulnay-sous-Bois, France; 3 CentraleSupelec-L2S-Laboratoire des signaux et systèmes, Brain and Spine Institute, Université Paris-Sud, Orsay, France; 4 Human Biomonitoring Research Unit, Luxembourg Institute of Health, Strassen, Luxembourg; 5 L’Oréal Research and Innovation, Pudong, China; 6 L’Oréal Research and Innovation, Singapore, Singapore

**Keywords:** Skin microbiota, Sequencing, Urban pollution, Polycyclic aromatic hydrocarbons

## Abstract

**Background:**

Polycyclic aromatic hydrocarbons (PAHs) are of environmental and public health concerns and contribute to adverse skin attributes such as premature skin aging and pigmentary disorder. However, little information is available on the potential roles of chronic urban PAH pollutant exposure on the cutaneous microbiota. Given the roles of the skin microbiota have on healthy and undesirable skin phenotypes and the relationships between PAHs and skin properties, we hypothesize that exposure of PAHs may be associated with changes in the cutaneous microbiota. In this study, the skin microbiota of over two hundred Chinese individuals from two cities in China with varying exposure levels of PAHs were characterized by bacterial and fungal amplicon and shotgun metagenomics sequencing.

**Results:**

Skin site and city were strong parameters in changing microbial communities and their assembly processes. Reductions of bacterial-fungal microbial network structural integrity and stability were associated with skin conditions (acne and dandruff). Multivariate analysis revealed associations between abundances of *Propionibacterium* and *Malassezia* with host properties and pollutant exposure levels. Shannon diversity increase was correlated to exposure levels of PAHs in a dose-dependent manner. Shotgun metagenomics analysis of samples (*n* = 32) from individuals of the lowest and highest exposure levels of PAHs further highlighted associations between the PAHs quantified and decrease in abundances of skin commensals and increase in oral bacteria. Functional analysis identified associations between levels of PAHs and abundance of microbial genes of metabolic and other pathways with potential importance in host-microbe interactions as well as degradation of aromatic compounds.

**Conclusions:**

The results in this study demonstrated the changes in composition and functional capacities of the cutaneous microbiota associated with chronic exposure levels of PAHs. Findings from this study will aid the development of strategies to harness the microbiota in protecting the skin against pollutants.

Video Abstract

## Background

The human skin hosts a diverse community of commensal bacteria, fungi, viruses, and parasites, which collectively constitute the skin microbiota. The skin microbiota is indispensable for host skin health, as dermatological conditions have been linked to the alteration of the cutaneous microbiota [1–3]. Recent developments in the field have provided insights into how the cutaneous microbiota is influenced by physiological, anthropogenic, and environmental factors [4–6]. In particular, the proximity of the cutaneous microbiota to the external environment raises questions about the degree to which the environmental exposome can modify these microbes, and how such modifications may affect skin health.

Environmental pollution is now a global concern, as the World Health Organization (WHO) estimates that over seven million people die annually due to illnesses attributable to both ambient and indoor air pollution exposure [7]. Atmospheric pollution is a particularly dire issue in China, where rapid industrialization has led to alarming levels of pollutants across cities. Clinical studies have correlated daily exposure to particulate matter (PM) with increased skin pigmentation spots and wrinkles [8–10]. Among the constituents of harmful atmospheric pollution, polycyclic aromatic hydrocarbons (PAHs), a class of organic pollutants in ambient air commonly associated with PM, may enter the human body and bloodstream via inhalation, ingestion, and dermal absorption [11–13]. While chronic dermal exposure to PAHs may be associated with premature skin aging, pigmentary disorder, acne, and skin cancer [10, 14, 15], the exact mechanisms by which PAH pollution harms the skin remain poorly understood and are likely to be complex and multifaceted.

**Fig. 1:**
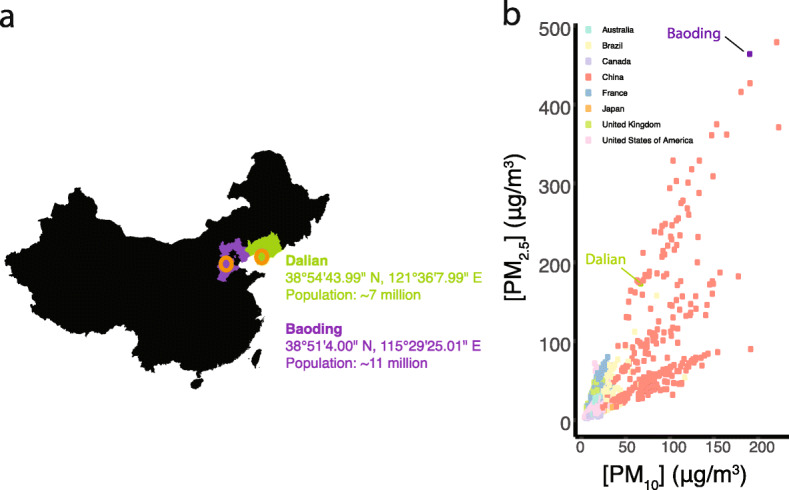
Cohort city characteristics. **a** Skin microbiota from individuals living in the cities of Baoding and Dalian in China were characterized. City population figures obtained from Palazzi et al. [11]. **b** Annual mean concentrations of PM_10_ and PM_2.5_ in outdoor air for cities of selected countries for the years 2013 to 2016. Concentration measurements for Baoding and Dalian are highlighted. PM_10_ and PM_2.5_ data acquired from the WHO (http://www.who.int/airpollution/data/cities/en/, accessed 5th November 2018)

Recently, bacterial skin commensals have been demonstrated to degrade different PAHs and related xenobiotic compounds [16, 17]. Such reports demonstrate a potential ecological connection between PAH exposure and the microbiota in skin disorders. Given the health-related risks of PAH exposure, the importance of the skin microbiota and the ability of skin commensals to metabolize PAHs, the roles PAHs have in the cutaneous microbiota deserve to be comprehensively examined. Therefore, using amplicon- and metagenomics-based sequencing, cheek and scalp microbiota of 204 individuals residing in two cities in China with different levels of exposures to PAHs and related pollutants, one heavily polluted (Baoding) and the other less so (Dalian) (Fig. 1), were characterized with a focus on the potential roles of pollutant exposure on shaping the skin microbiota. The results from this study demonstrate that differences in pollutant exposure levels have profound effects on the skin microbial community composition in a dose–effect manner, and on their functional potentials that may be important for microbiota-skin homeostasis.

## Results

### Skin site and city as main drivers of microbiome variation in study population

Participants living in the two cities were evaluated for their facial signs and exposure to PAHs in 12-cm hair samples (reflecting the extent of exposure during a 1-year period) [10, 11]. An increased severity was observed in almost all facial signs, including wrinkles and pigmentation disorders, in individuals living in Baoding [10]. Furthermore, biomonitoring of PAHs showed quantitative differences in the exposure levels between the two populations [11]. Specifically, of the parent PAHs and metabolites detected, 14 out of 15 and 14 out of 56 presented significantly higher concentrations in the hair samples of subjects from Baoding compared to Dalian, respectively. In Baoding, the median concentration was 1.5 to 2.8 times higher for parent PAHs and 1.1 to 2.3 times higher for metabolites than in Dalian. Among the parent PAHs quantified, higher levels were observed for phenanthrene, fluoranthene, pyrene, fluorene, acenaphthylene, and anthracene, while for metabolites, 9-OH fluorene, 2-OH-naphtalene, and 1-OH-anthracene were higher [11].

**Fig. 2:**
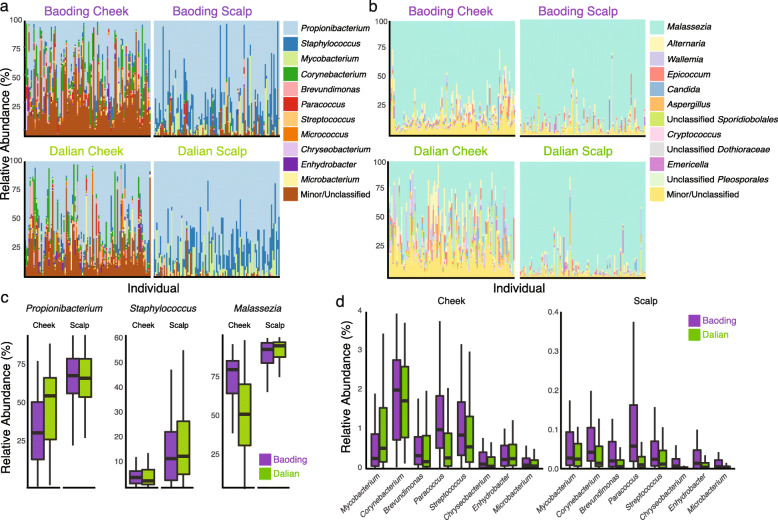
Taxonomic overview of the skin microbiota. Major (**a**) bacterial and (**b**) fungal genera detected on skin and scalp sites in Baoding and Dalian. **c** Relative abundances of common colonizers *Propionibacterium*, *Staphylococcus*, and *Malassezia* across cities and sites. **d** Relative abundances of other common human-associated and environmental genera detected across cities and sites

Amplicon sequencing provided an overview of the cheek and scalp microbiota profiles of individuals residing in Baoding and Dalian (Fig. 2a, b). The skin site was a major driver of taxonomic variation. Greater relative abundances of *Propionibacterium* (false discovery rate (FDR)-adjusted *p* = 8.8×10^−16^), *Staphylococcus* (FDR-adjusted *p* = 8.8×10^−16^), and *Malassezia* (FDR-adjusted *p* = 6.6×10^−16^) were detected on scalps than on cheeks (Fig. 2c). *Enhydrobacter*, a bacterial genus thought to be enriched in Chinese individuals [18–20], was ten times more abundant on cheek sites (FDR-adjusted *p* = 8.8×10^−16^) compared to the scalp. Other top genera included skin colonizers with presumptive human and environmental origins (Fig. 2d). Here, we show that city was an additional main driver of taxonomic variations. Communities from Dalian showed a higher abundance of *Propionibacterium* and a greater diversity of sub-genus oligotypes (OGTs) within *Propionibacterium*, namely an increased relative abundance of OGT02 (relative abundance of 2.0% and 24.0% in Baoding and Dalian, respectively, Additional file 1: Figure S1a, FDR-adjusted *p* = 8.8×10^−16^). In contrast, distributions of *Staphylococcus* and *Corynebacterium* OGTs appeared to be body site-specific, with *Staphylococcus* OGT01 more abundant on scalps (19.4% and 72.2% on cheeks and scalps, respectively, Additional file 1: Figure S1b, FDR-adjusted *p* = 8.8×10^−16^), and *Corynebacterium* OGT01 more abundant on cheeks (21.4% and 12.8% on cheeks and scalps, respectively, Additional file 1: Figure S1c, FDR-adjusted *p =* 2.8×10^−10^).

Differential abundance analysis was performed to highlight city differences in the enrichment of operational taxonomic units (OTUs) or taxa, associated with adverse skin phenotypes (healthy vs. acne on the cheek and healthy vs. dandruff on the scalp, Additional file 2: Figure S2). Taxa of the same genera were found differentially abundant in the two cities (e.g., OTU_B5 and B10 of *Corynebacterium*, OTU_B11095 and B2738 of *Staphylococcus*, and OTU_F4802 and other OTUs of *Malassezia* were over-represented in Baoding and Dalian, respectively). *Micrococcus*, *Paracoccus*, *Ralstonia*, *Novosphingobium*, and *Aestuariimicrobium* (Additional file 2: Figure S2), genera that have been documented to break down PAHs and related compounds [16, 21–24], were among the taxa enriched in Baoding independent of the skin or scalp type. The majority of the differentially abundant fungal taxa were enriched in Dalian.

Microbial diversity (i.e., alpha-diversity) analysis showed that cheeks were more diverse than scalps for both bacteria and fungi (Additional file 3: Figure S3a), and there was a higher diversity in the cheek samples from Baoding compared to Dalian samples (FDR-adjusted *p* = 0.0002 for both bacteria and fungi). In terms of skin type, individuals from Baoding with acne presented a slightly lower bacterial diversity on cheek compared to healthy individuals, while acne had no association with bacterial diversity in Dalian individuals nor an association with lower fungal diversity in either city (Additional file 3: Figure S3b). Scalp samples did not differ in diversity between healthy or dandruff-affected individuals (Additional file 3: Figure S3c).

As the microbial community compositions (i.e., beta-diversity) were significantly different between skin sites (Additional file 4: Table S1), community dissimilarity was analyzed for each site and skin type. For cheeks, the city was a significant clustering factor for both bacterial and fungal communities regardless of the metric used (Additional file 4: Table S1), whereas for the scalp, the bacterial composition (weighted UniFrac distance) was not different between cities (Additional file 4: Table S1). In Baoding only, individuals with acne presented different bacterial community compositions on cheeks, as compared to healthy subjects (Additional file 4: Table S1). Similarly, bacterial community composition on dandruff-affected scalps in Baoding clustered away from healthy scalps (Additional file 4: Table S1) and was marginally significant when classified according to dandruff severity (Additional file 4: Table S1). The age of the participants was a significant factor when explaining fungal communities on cheeks in Baoding but not in Dalian. Scalp fungal community memberships and compositions within each city did not appear to be influenced by age or dandruff symptoms. No significant effect of age was observed on the bacterial community. Overall, these results suggest complex interplays between how skin site, cohort location, and skin type potentially affect the skin microbial community structure.

### Acne and dandruff were associated with reduced integration and stability in their cross-domain association networks in Baoding and Dalian

Recent optimization of correlation network algorithms has enabled the analysis of inter-domain associations [25]. Here, using bacterial and fungal data of the 204 subjects, we applied correlation networks to explore whether (1) the microbial association network structural properties differed between cheek and scalp sites of the two cities, and (2) the microbiota of acne and dandruff sites showed changes in network stability compared to healthy sites in Baoding and Dalian.

**Fig. 3:**
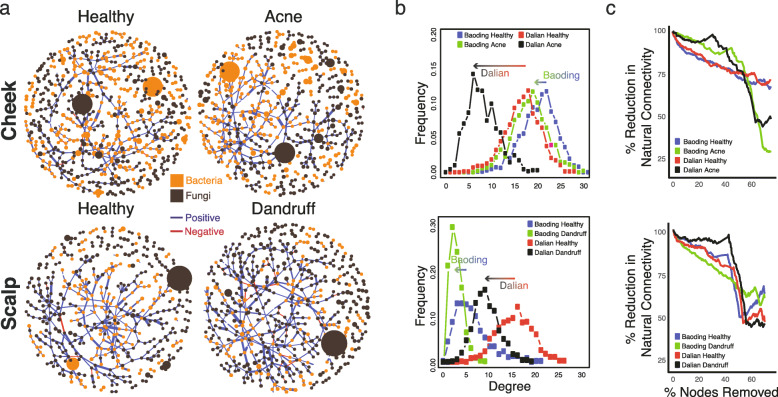
SPIEC-EASI cross-domain correlation network analysis of the skin microbiota. **a** Overall cross-domain network structure for the cheek and scalp sites (city combined) and skin condition. Taxa are represented by nodes and shaded by their domain (bacteria in orange, fungi in brown). Blue and red edges represent positive and negative correlations, respectively. Node size and edge thickness refer to OTU abundance and correlation strength, respectively. **b** Node degree distribution of networks for cheek and scalp microbiota, grouped by city and skin condition. Arrows indicate the decrease in peak degree distribution from healthy to affected skin sites for the respective cities. **c** Structure stability for microbial networks of cheek and scalp sites of different cities and skin phenotypes. Decrease in network stability is represented by percentage reduction of natural connectivity upon betweenness-ranked node attack removal

The majority of the inter-domain associations detected here were positive regardless of city, site, or site phenotype (acne/dandruff vs. healthy, Fig. 3a, Additional file 5: Figure S4, and Additional file 6: Table S2). Central nodes were not necessarily the most abundant OTUs, suggesting that the roles of taxa in network associations were independent of abundance. Some of the strongest positive correlations were observed between a wide range of bacteria and fungi, while the majority of negative correlations involved *Malassezia* (Additional file 6: Table S2). Importantly, taxa of *Malassezia* and those of *Propionibacterium* and *Staphylococcus* were involved in both positive and negative cross-domain correlations (Additional file 6: Table S2). For both cities, microbiota networks on cheeks with acne and scalps with dandruff were less integrated than their healthy counterparts, with lower average node degree (Fig. 3b) and decreased network stability upon node attack removal (Fig. 3c), reminiscent of a previous work documenting the network collapse of dandruff-affected sites [2]. This shift in average node degree was lower in Baoding than in Dalian in both skin and scalp conditions. Nonetheless, our observations here show that microbial network fragility may be a general phenomenon associated with adverse skin conditions.

### Bacterial and fungal taxa showed significant associations with exposure levels of PAHs and skin parameters independent of skin site and city of origin

In addition to city differences, the analysis of PAHs in the hair samples by Palazzi et al. [11] revealed that each person had a PAH pollution profile composed of diverse PAHs (parent and metabolites) at various concentrations averaged over a 1-year exposure period. Therefore, instead of using one single PAH as a biomarker, we defined an index of the pollution exposure intensity using a principal component analysis (PCA)-based approach to delineate eight groups of subjects with an increasing score of pollution in order to further investigate the PAHs dose-response [26] (Additional file 7: Figure S5a-b and Additional file 8: Table S3). When cheek samples were considered, we observed negative and positive correlations between PAH exposures and relative abundances of OTUs classified as *Propionibacterium* (Spearman’s rho = −0.162, *p* = 0.022) and *Malassezia* (Spearman’s rho = 0.148, *p* = 0.035) respectively, suggesting a dose-dependent response between the PAH exposure levels and the abundances of these two major skin colonizers. Scalp sites showed a similar trend for *Malassezia* (Spearman’s rho = 0.143, *p* = 0.0041) but not *Propionibacterium* (Spearman’s rho = 0.0479, *p* = 0.50). A shift in overall taxonomic composition was also observed along with the different pollution exposure groups (Additional file 7: Figure S5c-d). In addition, samples from individuals with increasing levels of PAHs presented greater Shannon bacterial diversity on check and scalp (Additional file 7: Figure S5e-f), whereas positive correlations of fungal diversity across the exposure groups was significant only on scalp (Additional file 7: Figure S5g-h).

Sparse canonical correlation analysis (sCCA) was performed to explore global associations between relative abundances of OTUs and overall PAH pollutant exposure. Significant correlations were observed between exposure levels of PAHs and 23 bacterial (Additional file 9: Figure S6a and Additional file 10: Table S4) and 21 fungal (Additional file 9: Figure S6b and Additional file 10: Table S4) OTUs. In order to obtain a representation of the subjects in a common consensus space (compromise space of latent components that integrated either bacterial and PAH modalities or fungal and PAH modalities), a hierarchical multi-block analysis (MAXVAR-A) based on variables selected from the sCCA was carried out (Additional file 9: Figure S6c-d). The consensus representation exhibited clear grouping between samples from the two cities, even though the city of origin was not included in the analyses, suggesting correlations between the presence of pollutants and microbial taxa on the skin.

Multivariate association with linear models (MaAsLin2) was performed to further assess the relationships between OTUs and host factors including exposure levels to specific PAHs and related pollutants while accounting for the effects of covariates. Various bacterial and fungal OTUs were significantly associated with host’s age, city, and presence of acne or pigmentary disorder frequency for cheek and dandruff severity for scalp samples (Additional file 11: Table S5). Interestingly, the majority of the significant associations detected (~60%) were those between the relative abundances of particular OTUs and exposure of specific PAHs and related compounds (Additional file 11: Table S5). Among the significant correlations, dandruff severity was correlated to 5 *Malassezia* OTUs in scalp samples, which is similar to a previous study [27], while in cheek samples, a few bacterial and fungal OTUs were correlated to acne (*n* = 2), age (*n* = 7), and pigmentary disorder frequency (*n* = 5).

### Majority of pollutant-associated signature taxa deviated from the neutral assembly process

**Fig. 4:**
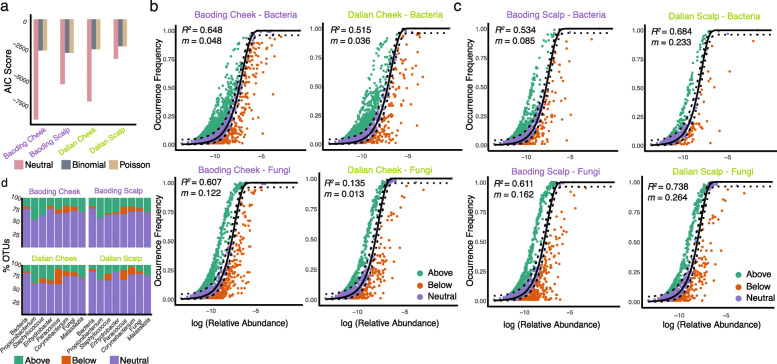
Sloan neutral model predictions of skin microbiota. **a** AIC score comparing the ability of the neutral, binomial, and Poisson models in explaining the skin microbiota assembly process. **b**, **c** Sloan neutral model prediction of (**b**) cheek and (**c**) scalp microbiota grouped by city. OTUs are represented by data point and colored according to whether the taxon fitted above (green), within (purple), or below (orange) the 95% confidence interval (dotted lines). *R*^*2*^ values (measurement of fit to neutral assembly process) and *m* values (estimated migration rate) are indicated for each prediction. **d** Proportion of OTUs within each of the main skin-associated microbial genera according to their Sloan neutral model prediction. Bars labeled “bacteria” and “fungi” represent the overall proportion of all taxa within the respective domains

A recent study demonstrated that the degree of urbanization in a city may be associated with differences in the accuracy of the Sloan neutral model in predicting the microbial assembly process on cheeks [28]. Here, we tested whether both the cheek and scalp microbiota in our cohort can also be explained by the neutral model. As demonstrated by the lower Akaike information criterion (AIC) score, the neutral model was more suitable in predicting the assembly of the skin microbiota in our cohort compared to the binomial and the Poisson models (Fig. 4a). For both bacteria and fungi, cheek sites from the more urbanized city of Dalian showed a greater tendency for niche-based assembly compared to the more industrial city of Baoding (as indicated by the lower goodness-of-fit *R*^2^ values and migration rates *m* on cheeks in Dalian, Fig. 4b). In contrast, the scalp sites showed the opposite trend, where Baoding showed a greater tendency for niche-based assembly (Fig. 4c), suggesting that both city and skin site contribute to the ability of the neutral model to predict skin microbial assembly.

The majority of the OTUs fell within the 95% confidence interval of the neutral model prediction (Fig. 4d and Additional file 12: Table S6). Skin colonizers including *Propionibacterium*, *Staphylococcus*, and *Malassezia* had greater proportions of their OTUs presenting above the prediction model compared to the overall bacterial and fungal communities (Fig. 4d), indicating that these genera contained higher proportions of their OTUs at a greater prevalence than expected by the model given their abundance. Conversely, for both cities, *Paracoccus* and *Corynebacterium* had greater proportions of their OTUs presenting below the model prediction, indicating that these genera contained higher proportions of their taxa detected in fewer samples than expected. Different bacterial and fungal OTUs of common skin genera (*Propionibacterium*, *Corynebacterium*, *Staphylococcus*, *Malassezia*) could be consistently detected above or below the prediction model regardless of city and site (Additional file 13: Table S7). Also, a large number of OTUs of unknown genus deviated from the model prediction in Baoding regardless of site or within scalp sites regardless of the city (Additional file 13: Table S7). The OTUs showing significant associations with exposure levels according to MaAsLin2, in the majority, deviated from the neutral model prediction (Additional file 11: Table S5).

### Associations between exposure levels of PAHs and commensal bacteria on skin revealed by pilot metagenomics

**Fig. 5:**
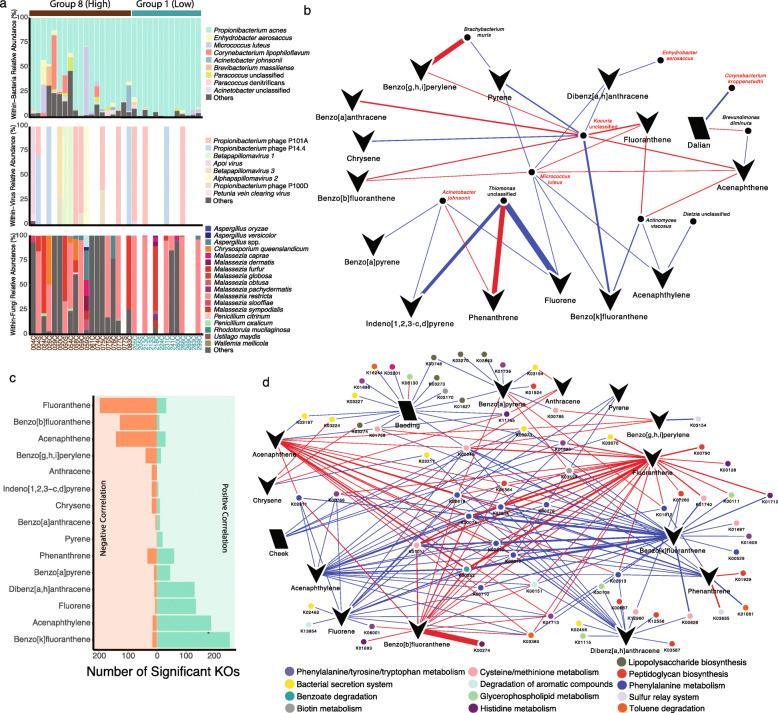
Species-level taxonomic and KO analysis of potential functions fof the skin microbiota based on metagenomics sequencing. **a** Bacterial, viral, and fungal species distribution of 32 individuals. In cases where bars were not present, the corresponding domains were not detected in the samples according to the classification methods employed. Sample names on x-axis are colored based on whether they come from individuals from the lowest (Group 1, turquoise) or highest (Group 8, brown) of PAH exposure groups. **b** Correlation network depicting significant associations between city, skin site and/or PAH exposure levels to the relative abundance of species. Blue and red edges represent positive and negative correlations, respectively. Species in red indicate those considered to be part of the commensal skin flora. The thickness of edges represent the magnitude of correlation. Additional file 15: Table S8 shows the correlation magnitude and *q* values for all significant taxonomic associations, including those with additional host factors not shown here. **c** Number of KO gene families showing significant correlations with exposure to different PAHs. **d** Correlation network showing significant correlations between city, skin site and/or PAH exposure levels to the relative abundance of KOs of selected pathways. Blue and red edges represent positive and negative correlations, respectively. The thickness of edges represent the magnitude of correlation. Additional file 16: Table S9 shows the correlation magnitude and *q* values for all significant functional associations, including those with additional host factors not shown here. For both taxonomic and functional analyses, FDR-adjusted *p* value (*q* value) ≤ 0.25 is considered significant as determined by MaAsLin2

A pilot metagenomics analysis involving samples from representative individuals of the lowest and highest PAH exposure groups (Additional file 7: Figure S5a) was undertaken to further evaluate the species-level taxonomic and functional relationships of the skin microbial communities upon PAH exposure, which could not be assessed by amplicon sequencing. From the 32 samples included in the analysis (*n* = 13 samples for lowest PAH exposure group, *n* = 19 samples for highest PAH exposure group), the majority of reads were assigned to bacteria (average relative abundance 88.5%), followed by virus (10.2%), fungi (1.25%), and archaea (0.005%) (Additional file 14: Figure S7). Detection of species within the genera *Propionibacterium*, *Corynebacterium*, *Staphylococcus*, *Paracoccus*, and *Enhydrobacter*, which were among the most prevalent and abundant genera in amplicon analysis, was confirmed by metagenomics (Fig. 5a). *Propionibacterium acnes* (recently classified as *Cutibacterium acnes* [29]) phage P101A/P101D and *Betapapillomavirus type 3* were the most common DNA viruses detected (Fig. 5a), in line with previous reports showing the dominance of viruses including *P. acnes*-phages on the skin [30]. The cheek site of one individual (50C) had a high relative abundance of *Betapapillomavirus type 3* (86.6% of the entire microbial community detected). Multiple species of *Malassezia*, including *M. restricta* and *M. globosa*, were some of the most abundant fungi detected (Fig. 5a).

MaAsLin2 analysis on metagenomics taxonomic data revealed an association between specific levels of PAHs on cheeks with skin commensals *Micrococcus luteus*, *Acinetobacter johnsonii*, *Enhydrobacter aerosaccus*, as well as an unclassified *Kocuria* species (Fig. 5b and Additional file 15: Table S8). In particular, significant negative correlations were observed between *M. luteus* and fluoranthene and benzo[b]fluoranthene, while a significant negative correlation was detected between *A. johnsonii* and phenanthrene, suggesting that exposure to these PAHs may result in a reduction of commensal colonizers and any potential protective effects provided by these commensals against pathogen colonization. In addition, benzo[k]fluoranthene and acenaphthylene showed significant positive associations with abundances of oral bacteria *Actinomyces viscosus* (average relative abundance 0.006%).

### Exposure to specific PAHs associated with different functions involved in microbial-host interactions

Functional potential profiling of the skin microbiota identified 721,709 UniRef50 gene families grouped into 6969 KEGG Orthology (KO) families. Within these families, nearly 1746 KOs were identified by MaAsLin2 as showing significant associations with skin site (25 associations), city (139 associations), and/or exposure levels to particular PAHs (1582 associations), encompassing genes involved in a variety of microbial metabolic, signaling, and information processing functions (Fig. 5c, d and Additional file 16: Table S9). The number of KOs associated with each PAH quantified did not appear to be governed by the molecular weight and structure of the PAHs. Exposures to some PAHs (e.g., phenanthrene, benzo[k]fluoranthene, acenaphthylene, fluorene, pyrene, etc.) were mainly positively associated with the relative abundances of multiple KOs, while exposures to other PAHs (fluoranthene, benzo[b]flouranthene, and acenaphthene) resulted in an overall negative correlation with the relative abundances of multiple KOs (Fig. 5c, d), suggesting that exposures to different PAHs may be linked to differential microbial functional responses.

KOs associated with exposure levels of PAHs, according to MaAsLin2 analysis, included mainly those belonging to microbial metabolic pathways linked to (i) host physiology including biosynthesis of amino acids and biotin metabolism, (ii) microbial virulence including bacterial secretion systems, glycerophospholipid, and lipopolysaccharide biosynthesis, and (iii) aromatic compounds degradation (Fig. 5d). For amino acid metabolism, pathways involved in tryptophan metabolism (K00274 monoamine oxidase and K00128 aldehyde dehydrogenase) were negatively correlated to PAHs, while those related to biosynthesis (K00766 *trpD*, K01609 *trpC*, K01695 *trpA*, K01696 and K06001 *trpB*) of tryptophan were both positively and negatively associated with exposures to different PAHs. Similar tryptophan biosynthesis pattern has been reported in *S. aureus* isolates and metagenomes from atopic dermatitis lesions [31, 32]. Other amino acid metabolism pathways (histidine, cysteine, and methionine metabolism), which have been previously related to dandruff scalp [33], were also associated with exposure levels of certain PAHs.

Abundances of KOs encoding virulence factors such as secretion systems (K03197 VirB2 and K03194 VirB1 [34]) and peptidoglycan biosynthesis (K00790 *murA*, K01924 *murC*, K01929 *murF*, K05364 *pbpA*, K12556 *pbp2X*, and K00687 *pbp2B* [35]) were also observed to be correlated with exposure levels of some PAHs. Specifically, KOs related to lipopolysaccharide synthesis pathways, which are crucial for bacterial virulence [36], were significantly associated with exposure to benzo[a]pyrene.

Genes belonging to pathways related to PAHs degradation such as *gyrA* (K00015) and *fdhA* (K00148) [37] were significantly associated with exposure levels of acenaphthylene, benz[k]fluoranthene, and fluorene, in addition to KOs of pathways important for the degradation of aromatic and xenobiotic compounds such as benzoate and toluene (Additional file 16: Table S9). KOs related to phenylalanine metabolism (K02610 *paaB*, K02611 *paaC*, K02612 *paaD*, K00074 *paaH*, K02614 *paaI*, and K02618 *paaZ*), reported to be involved in the aerobic strategy of aromatic compounds degradation by bacteria [38], were also associated with increased exposure levels to multiple PAHs (Fig. 5d and Additional file 16: Table S9).

## Discussion

Among the constituents of atmospheric pollution, human exposure to PAHs and related pollutants in ambient air represents a universal public health issue, partly because of its association with undesirable effects on human skin [10, 14, 15]. This study highlighted the potential roles pollutant exposure have on various aspects of the skin microbiota, including changes in diversity and abundances of taxa and alteration of the functional potentials that may be important for the commensal microbiota to protect the host against pathogens and maintain skin homeostasis. Given the clinical relevance of pollutant exposure to the skin conditions, and the PAH biodegradation properties of resident skin microbes [16, 17], examinations of the interplay between PAH exposure and skin microbiota enable a first understanding of the impacts PAHs and related pollutants have on cutaneous health.

Consistent with previous works [4–6, 28], this study showed that skin site and the cohort residing city played major roles in shaping bacterial and fungal cutaneous communities. In particular, a diversification of the microbiota was observed along with a reduction in *Propionibacterium*. The diversification also included the enrichment of taxa belonging to genera with biodegradation potentials in the more polluted city of Baoding. While some of these genera were detected as rare members of the skin microbiota in this study and in previous reports [39–41], their ability to degrade PAHs [21, 42] warrants further attention.

Our network analysis provided a representative estimation of potential ecological relationships by incorporating cross-domain data [25]. Reduced network structure connectivity and stability on scalp sites affected with dandruff are consistent with a previous study comparing network structures between healthy and dandruff-affected scalps [2]. Here, we showed that acne-affected cheeks also showed reduced network structural stability, suggesting that reduced microbial network integrity is a general property associated with skin disorders, or conversely that a robust and stable network may be protective of cutaneous health. While our study was not able to compare network features between individuals with varying exposure levels of different pollutants, future studies focusing on the roles of PAHs in potentially altering microbial network characteristics may provide solutions for alleviating any adverse effects of PAHs on skin physiology.

Skin sites and geography influenced how well the neutral model fitted bacterial and fungal community assemblies. As demonstrated by Kim et al. [28], cheek microbiota of individuals from urban centers were better fitted to a niche-based assembly model. Our observations on cheek sites were consistent with this notion. In contrast, the scalp data showed that the assembly process of the microbiota in the less urbanized city of Baoding was better fitted to the niche-based model. Taken together, the cheek and scalp data suggest that assemblies of the bacterial and fungal communities may be governed by both the environment and skin site (and possibly additional factors not examined). This is perhaps expected, as the microbial assembly is a dynamic process likely to be dependent on both the environment and host physiology, with the latter known to differ by skin sites [4]. Different taxa within common skin genera (i.e., *Propionibacterium*, *Staphylococcus*, *Corynebacterium*, and *Malassezia*) can both fit and deviate from the neutral model prediction, suggesting within-genera differences in colonization potential and extending previous findings of varying cutaneous colonization and pathogenic capabilities between related species and strains [3, 43]. While most of the taxa in the community fit the neutral model prediction, the majority of the taxa associated with pollutant exposure levels deviated from the prediction, indicating that these microbes may be selected (for or against) among the populations.

**Fig. 6:**
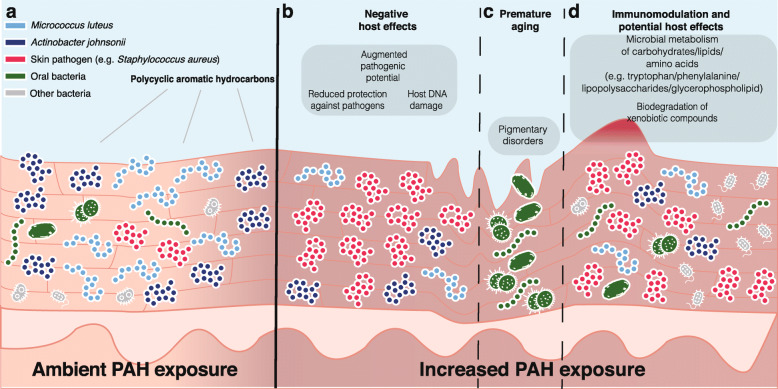
Schematic diagram showing potential roles of PAH exposure on skin microbiota and skin phenotype. **a** Exposure of PAHs from environmental and anthropogenic sources. **b** PAH exposure levels are associated with changes in abundances of skin colonizers, which may change their protective abilities against potential skin pathogens, as well as host DNA damage. **c** PAHs can also affect the levels of oral species, which may play roles in altered skin phenotypes including wrinkle formation and psoriasis. **d** Changes in PAH levels are also associated with changes in abundances of functional genes of the microbiota, which may render the skin prone to changes in epidermal physiology and its ability to degrade pollutants

City-associated differences in community composition, assembly, and network structures described above cannot be fully attributed to city-based differences in PAH exposure. However, multivariate analyses directly identified associations between levels of PAHs and microbial members and their functional profiles. Based on the observations presented, we propose a conceptual mechanism of the potential roles that PAHs have on the skin microbiota and cutaneous health (Fig. 6). PAHs are brought to the skin either by direct contact and exposure or via systemic bloodstream following inhalation or ingestion (Fig. 6a) [11–13]. Regardless of the delivery mode, our results demonstrate that detected levels of PAHs were associated with shifts in abundances of commensal species (Fig. 6b). Specifically, we detected negative correlations between fluoranthene and benzo[b]fluoranthene and relative abundance of the skin commensal *M. luteus*. Given the potential protective effects of *M. luteus* against ultraviolet (UV)-induced DNA damage on the skin [44], our observations raise the possibility that PAH-associated DNA damage [45] could be partially be mediated by changes in the skin microbiota. The pilot study described here on a relatively small set of skin microbiota metagenomes did not detect significant relationships between PAH exposure and the abundance of another commensal *Staphylococcus epidermidis*. *S. epidermidis* protects the host against the colonization by external species and the development of pathogenic traits in hosts [46], in addition to facilitating wound healing in hosts [47] and protecting against the growth of skin tumors [48]. Given the number of protective effects, this commensal exerts on skin physiology, a more thorough and expanded investigation will allow us to examine whether PAH exposure is also linked to the abundance of this protective commensal.

Besides changes in exposure levels of different PAHs associating with variations in abundances of commensals skin colonizers, there is also a positive relationship with the cutaneous abundance of oral bacteria potentially linked to atopic dermatitis (Fig. 6c) [1]. More significantly, oral bacteria have been implicated in skin aging in Asians over the age of 60 [49], and the observed positive correlation between exposure levels of PAHs and abundance of oral bacteria, therefore, highlights one potential mechanism by which PAHs contribute to skin disorders and aging via the accumulation of oral bacteria on the skin. It is worth noting that the current study involved subjects ≤ 45 years old, which were younger than the cohort in Shibagaki et al. [49]. Therefore, whether the accumulation of oral bacteria on the skin in younger individuals leads to premature skin aging deserves further attention in the future.

Detected levels of PAHs were associated with changes in abundances of microbial genes encoding various metabolic functions (Fig. 6d), consistent with a recent study demonstrating alterations in the proteome of soil microbiota following pollutant exposure [50]. Interestingly, genes involved in the metabolism of amino acids and vitamins, which may be important in the maintenance of healthy scalps [33], showed negative correlations with exposure levels of some PAHs. This is consistent with the potential effects PAH exposure have on increased susceptibility to skin conditions (e.g., dandruff formation) via reducing the microbiota’s potential to process amino acids and vitamins [33]. Also, exposure levels to some PAHs were significantly correlated with genes encoding tryptophan metabolism, which has been shown to be important for modulating host-microbial cross-talk [51]. It must be noted that, unlike as demonstrated in the human gut [51], there is scarce information on whether metabolites of these microbial pathways that appeared to be associated with levels of PAHs play roles in host skin physiology [52]. However, given that differences in abundances of microbial genes of the tryptophan pathway are associated with AD [1], our observations pave the way for future studies in exploring the potential roles PAH exposure have in affecting potential interactions between host and the skin microbiota.

Positive correlations between exposure levels of PAHs and abundances of species detected in our pilot study are consistent with PAHs being carbon and energy sources for these taxa [17] (Fig. 6d). While degradation of PAHs to benign products by skin microbiota can potentially eliminate the detrimental effects of PAHs on the skin [16], partial metabolism of PAHs may yield a wide variety of intermediate metabolites that may be toxic to host tissues and DNA [17]. Given that the cutaneous microbiota is strongly governed by the physiochemical properties of the skin surface [4], greater insights into the chemical makeup of the skin following complete or partial degradation of PAHs and other pollutants will be beneficial in understanding how PAH metabolism affects skin health through modulating microbial compositions and functions. Specifically, whether changes in abundances of these functional genes by PAHs are due to shifts in the microbial community, and/or an adaptive response of the existing microbiota, remain to be elucidated.

Following the pilot shotgun metagenomics assessment in this study, future works involving a larger sample size will undoubtedly provide a more in-depth account of the potential roles PAH exposure have on the compositional and functional potential of the cutaneous microbiota. In addition, while a preliminary assessment was attempted in determining the viral community of the dataset, and some reads from the sebaceous cheek and scalp sites were classified as *Propionibacterium* phages, our DNA-based approach did not allow assessment of the RNA-viral populations. Subsequent works with laboratory and analytical methodologies specialized for virome analyses [53] will undoubtedly provide a more comprehensive account of the skin viral population, which is a crucial part of the cutaneous microbiota that links to host skin physiology [4] and acne [54]. Furthermore, metabolic functions of the skin microbiota working in a consortium to degrade PAHs, as seen in other ecosystems [37], can be linked to species-level taxonomical information via future metagenome-assembled genomic analyses.

## Conclusions

This study revealed a profound modification of the skin microbiota under chronic exposure to PAHs, in a dose–effect manner. Our data further found a reduced network structure connectivity and stability on affected skin and scalp sites with acne and dandruff respectively in the context of higher pollution exposure. This shift in the microbiota also resulted in modification of the microbiome functional potential linked to carbohydrates/lipids/amino acids metabolism, augmented pathogenic potential, and aromatic compounds degradation, which could be involved in the exacerbation of skin disorders observed in cities with air pollution. The findings presented here provide a first step towards a comprehensive understanding of the skin microbiota of individuals from cities with different ambient levels of PAHs. Given the continuous increased exposure of PAHs in almost all citizens in urban cities, future works adopting multidisciplinary approaches are required to elucidate the chemical, microbiological, physiological, and clinical repercussions of PAH exposures on the skin, thereby safeguarding our cutaneous health.

## Materials and methods

### Characteristics of the subjects and cities and sample collection

The research protocol was approved by the Sino-German Cosmetics Institute Ethics Review Board (Protocol 2015-033-DY-024) and was conducted according to the principles expressed in the World Medical Association Declaration of Helsinki. Informed written consent was obtained from all participants prior to any study-related procedure. The cohort included in this study was part of a larger series of works comparing the levels of PAHs in hair [11] as well as the association of PAH exposure with aging-associated facial signs [10] between the two cities. All participants provided information regarding health status, medical history, and daily habits. The participants had been living in the respective cities for at least 15 years, were non-smokers, did not receive antibiotics or systemic antifungals 1 month prior to sampling, did not have acute cutaneous disorders, nor had used depigmenting/whitening topical or systemic treatments 3 months prior to sampling, or exfoliating products 1 month prior to sampling. All volunteers had natural hairs from root to tip. In order to standardize the scalp condition, the participants were asked to wash their hair and scalp with the provided shampoo without anti-bacterial compounds for 2 weeks (three times per week) prior to sampling, and not to shampoo or apply any hair care or hair-styling product before sampling. Similarly, they were asked to wash their face with the provided neutral soap without anti-bacterial compounds for 3 days (once per day) prior to sampling. Last shampoo and soap were applied 48 and 24 h respectively before sampling. No other products were allowed on the scalp, hair, and face until sampling was performed.

All subjects of each city visited on a single occasion the facilities in Baoding and Dalian at different times in the morning during two successive weeks (Monday to Friday, approximately 20 visits per day). Cheek and scalp samples were collected from 204 healthy Chinese women, aged between 25 and 45 years, with 102 subjects residing in the relatively rural and industrial city of Baoding, a northern Chinese city in Hebei province recording high levels of atmospheric pollution, and 102 subjects residing in Dalian, an urbanized and modern northern Chinese city in Liaoning province with a lower degree of registered atmospheric pollution (Fig. 1a, b). The cities are located at the same latitude, share a similar climate and equivalent UV exposure (UV Index) over the last 15 years. Metadata for individuals and samples collected are provided in Additional file 17: Table S10.

Microbiota sampling was conducted in a climate-controlled room at 22 °C and 60% humidity. The samples for microbiome analysis were collected by using sterile cotton-tipped dry swabs that had been heated to 150 °C and pre-moistened with ST solution (0.15 M NaCl with 0.1% Tween 20). For cheek samples, swabs were rubbed firmly on the cheek for 60 s to cover a surface area of 2 cm^2^. For scalp samples, swabs were rubbed firmly on the scalp surface along a line by making four passages, then the swab was moved to another line by using a comb. These steps were performed six times to cover a total surface area of 4 cm^2^. After sampling, each cotton swab was placed into a microfuge tube and immediately flash-frozen in liquid nitrogen, and stored at −80 °C prior to genomic DNA (gDNA) extraction.

For skin physiological data collection, self-assessment questionnaire, clinical assessment, and skin measurements by dermatologists were conducted as described previously [10]. Scoring of skin signs and phenotypes was graded by dermatologists based on images as described previously [10, 55].

### Analysis of PAHs in hair samples

Exposure levels of PAHs can be determined through different approaches, such as assessment of pollutants concentration in environmental matrices (e.g., air, soil, dust) or in biological matrices (e.g., blood, urine, hair) [12, 13]. In this study, pollutant exposure levels were assessed via hair samples, providing results that are representative of the body exposure due to chemicals incorporated through biological pathways [11]. Briefly, hair samples were cut on the occipital area of each volunteer, as close as possible to the skin, and stored until pollutant measurement. For each subject, only the first 12 cm (starting from the skin) were analyzed. Considering a hair growth rate of 1 cm per month for most humans [56], the results thus correspond to 1 year of exposure prior to the time of hair sampling. The hair samples went through a decontamination procedure prior to analysis [57]. After hair pulverization, hydrolysis, and extraction, the extract was analyzed by GC-MS/MS or LC-MS/MS as described previously [11, 58–60]. The limits of detection for PAHs and metabolites of PAHs quantified were assessed as described previously [11]. PAH exposure levels for individuals included in the study are indicated in Additional file 17: Table S10.

### Grouping of subjects according to total PAH exposure levels

To investigate the relationships between exposure level to the collection of PAHs and the Shannon diversity index estimated on the cheek and scalp of individuals, the PAH measurements of all subjects were represented by a single score. This score was calculated by performing a principal component analysis (PCA) on log-normalized PAH measurements and keeping the first principle component (representing 36% of the total explained variance, PC1) as the new score variable. The score variable was then discretized into eight categories according to the distribution of the score variable to obtain eight balanced groups each containing a similar number of individuals, and with a distinct level of concentrations of PAHs between groups (Additional file 7: Figure S5a-b).

### Amplicon sample preparation for 16S rRNA gene and ITS sequencing

gDNA was extracted using the PowerSoil DNA isolation kit (MO BIO Laboratories, Carlsbad, CA, USA) following the manufacturer’s instructions with modifications as described previously [61]. In addition, following C6 elution, the elute passed through the same column filter an additional time to enhance yield. Negative controls of DNA-free water were extracted in parallel. Each gDNA sample was subjected to triplicate PCR by primers targeting the bacterial 16S rRNA gene V1-3 region, which is more accurate for depicting the skin bacterial community [62], and the ITS1 region as performed previously [5]. For both 16S rRNA gene and ITS1 analysis, amplicon-PCR and indexing-PCR were conducted on the 7500 Fast Real-Time PCR System (Applied Biosystems, Foster City, CA, USA), and amplicons were purified with the DNA/RNA Purification Beads (SeqMatic, Fremont, CA, USA). Library preparation and bacterial paired-end 300-bp and fungal paired-end 250-bp sequencing on the Illumina Miseq platform were performed by SeqMatic LLC (Fremont, CA, USA).

### 16S rRNA gene and ITS sequence processing and bioinformatics analysis

A total of 28,903,483 and 28,532,432 paired bacterial and fungal reads respectively in .fastq format were merged using the “-fastq_mergepairs” command in USEARCH (v9.2.64). Merged reads were filtered for quality control using the “-fastq_filter” command in USEARCH, with a maximum expected error rate of 0.01. Merged reads were trimmed to 450 bp and shorter reads were discarded. Filtered reads were subjected to OTU clustering at 97% sequence identity using the UPARSE [63] algorithm within USEARCH, and taxonomic information was provided for representative sequences of bacterial OTUs using the “assign_taxonomy.py” command in QIIME (v1.9) against the SILVA database (128 release). Fungal OTUs were interrogated against a previously curated fungal database designed for skin microbiome surveying [64]. Chimera detection was performed using UCHIME2 [65] within USEARCH under high-confidence mode. OTUs in taxonomic lineages present in greater than 5% of negative controls were deemed potential contaminants [19] and were removed from the dataset. In addition, chimeric, chloroplast, and mitochondria OTUs were also removed. Following quality control and removing undesirable reads, a total of 9,656,916 and 14,649,172 bacterial and fungal reads were retained, respectively. We verified that there was no bias in our experimental procedures by sequencing gDNA of artificial mixes of 10 bacterial and two fungal species (ZymoBIOMICS Microbial Community Standard, Zymo Research, Irvine, CA, USA), as well as in-house whole-cell controls containing mixture of varying proportions of known bacterial or fungal species (six different bacterial and five different fungal mixture controls were tested). The expected relative abundances were obtained in all the controls (data not shown).

Unless specified, the bioinformatics tools and methods employed applied to both bacterial and fungal sequences. Differential abundance analysis was performed using DeSeq2 available as the QIIME (v1.9) script “differential_abundance.py.” OTUs with fewer than 100 reads and present in < 25% of samples in each city (when comparing between sites within a city) or each skin site (when the comparison between cities within a site) were not included in analysis. Taxa with DeSeq2 log_2_ fold change ≥ |2| and false discovery rate (FDR)*-*adjusted *p* ≤ 0.05 were deemed significantly and differentially abundant. Oligotype analysis (pipeline v2.1) was performed for OTUs classified as *Propionibacterium*, *Staphylococcus*, and *Corynebacterium*. Specifically, all quality-filtered reads (not only representative reads) clustered into OTUs of the three genera were subjected to entropy analysis and oligotyping, with minimum substantive abundance criteria (-M) of 1000, 100, and 500 for *Propionibacterium*, *Staphylococcus*, and *Corynebacterium*, respectively.

Within-sample Shannon diversity was estimated using the breakaway (v4.0) package in R v3.5.1 [66]. The Good’s estimator of coverage was > 90% for all but one sample for both bacterial and fungal communities (Additional file 18: Table S11), indicating that the sequencing depth adopted was sufficient to capture the microbial diversity and richness. Beta-diversity analyses were performed to assess the community membership (unweighted UniFrac and Binary Jaccard distances for bacterial and normalized fungal datasets, respectively) and community composition (weighted UniFrac distance and Bray-Curtis dissimilarity for bacterial and normalized fungal datasets, respectively). Samples with total read counts below the normalized depth were excluded for alpha- and beta-diversity analyses.

To identify correlations between PAH exposure levels and bacterial and fungal taxa, pre-filtering was performed on OTUs based on relative abundance. OTUs with relative abundance < 0.1% across all individuals were removed. In addition, a cumulative sum scaling standardization (CSS) that corrects bias in the evaluation of differential abundance was applied. The CSS normalization method is an adaptive extension of the quantile normalization where raw counts are divided by the cumulative sum of counts up to a percentile determined using a data-driven approach. This approach proved to be more robust than the more commonly used total-sum scaling (TSS) normalization that has been shown to incorrectly bias differential abundance estimates in RNA-Seq data derived through high-throughput technologies [67]. Among the OTUs, 74 bacterial and 69 fungal taxa were selected after filtering for relative abundance. The PAH measurements were log-transformed to fit the Gaussian distribution. A total of 202 individuals with PAH and OTU data were included in the analyses. sCCA (a regularized version of the canonical correlation analysis used to study the relationship between two datasets while selecting only significant correlations [68]) was performed to select the OTU (bacteria or fungi) and PAH descriptors that were active in the between blocks relationships. The sparsity parameters of sCCA were selected with a permutation scheme using the CCA.permute function from the PMA (v1.0.9) package in R [68, 69]. The cross-correlation between selected PAHs and OTU relative abundance (bacteria or fungi) was visualized using a heatmap representation. Last, in order to obtain a common representation of individuals of the two blocks, a hierarchical multi-blocks analysis (MAXVAR-A) was performed using the RGCCA (v2.1.2) package in R [70].

To identify specific correlations between OTU relative abundances and PAH exposure levels while controlling for host covariates, MaAsLin2 [71] was performed separately on cheek and scalp samples. For both skin sites, age group, cohort city, and exposure levels to PAHs were included as covariates. In addition, for cheek analysis, host clinical parameters including acne onset, pore severity, clinical assessment of wrinkle grade, oiliness, and shininess on foreheads and cheeks, facial spot intensity, and frequency of pigment spots were also included as covariates. For scalp sites, scalp oiliness, hair volume, hair root dryness and greasiness, adherent dandruff scores, consumption of tap and purified water, and clinical assessment of dandruff status were included in analysis. Associations were considered significant if the FDR-corrected *p* value (*q* value) was ≤ 0.25.

### Cross-domain network analysis

SPIEC-EASI (SParse InversE Covariance Estimation for Ecological Association Inference, v1.0.4), modified for cross-domain analysis, was performed as described previously [25]. Networks were generated separately for Baoding and Dalian individuals with or without acne (for the cheek), and with or without dandruff (for the scalp). OTUs present in < 25% of the samples considered in each network analysis were excluded. SPIEC-EASI correlations ≤ |0.15| were excluded. The overall structure for each of the networks was assessed via degree distribution and natural connectivity in response to directed node removal based on decreasing node betweenness centrality.

### Sloan neutral model prediction

To assess whether the cheek and scalp communities for both cities exhibit either a neutral or a niche-based community assembly process, the Sloan neutral model analysis [72] was performed for each city and site as a group (i.e., total four groups) as described previously for the skin microbiota [28], with bacterial and fungal reads rarefied to 10,452 and 8,194 reads, respectively. Groups with a higher *R*^*2*^ value is consistent with the neutral microbial community assembly process, and the estimated migration rate (*m*) is a proxy of dispersal limitation. The fit of the neutral model was compared with the binomial and Poisson models based on their AIC scores. The Sloan model prediction and statistics were performed in R [28].

### Metagenomics sequencing library preparation and analysis

Thirty-two cheek and scalp samples were selected for metagenomics sequencing analysis. Samples from individuals in pollutant exposure groups one (lowest PAHs score group, *n* = 13) and eight (highest PAHs score group, *n* = 19) were selected (pollution exposure grouping set up as described above, Additional file 7: Figure S5a and Additional file 17: Table S10). gDNA of each sample was normalized to 10 ng/μL prior to library preparation. Library preparation was performed using standard Illumina protocol, and the final library was quantified with the 2200 TapeStation (Agilent, Santa Clara, CA, USA) and sequenced with the Illumina MiSeq platform by SeqMatic LLC (Fremont, CA, USA) to generate 150-bp pair-end reads.

Raw reads were subjected to adapter removal using AdapterRemoval (v2.2.2) [73], quality control and human DNA read removal using KneadData v0.6.1 (http://huttenhower.sph.harvard.edu/kneaddata), with reads shorter than 50 bp and reads mapping to the human reference genome hg38 discarded. Sequence quality information is provided in Additional file 19: Table S12. Taxonomic assignments of the filtered reads were performed using MetaPhlAn2 (v2.6.0, for bacterial and viral classification) [74] and FindFungi (v0.23.3, for fungal classification) [75]. Comparison of taxonomic classification between shotgun metagenomics and amplicon sequencing showed concordance (Additional file 20: Figure S8), consistent with previous works suggesting that the V1-3 region of 16S rRNA gene is appropriate for skin microbiota analyses [62]. Functional assignments were performed using HUMAnN2 (v0.11.1), with outputs expressed as reads per kilobase per million reads (RPKM). HUMAnN2 outputs were converted to KEGG Orthology (KO) gene families for heatmap representation and multivariate analyses of functional genes. MaAsLin2 was performed to associate PAH exposure to MetaPhlAn2 and HUMAnN2 data, with FDR-corrected *p* value (*q* value) ≤ 0.25 considered as significant as previously reported [76, 77]. Graphical network representations of significant MaAsLin2 correlations were designed using Cytoscape (v3.7.2) [78].

### Statistical analysis

Non-parametric Mann-Whitney test was performed to test for significance between two groups, while Kruskal-Wallis test was performed to test for significance between three or more groups. FDR-correction was performed using the “p.adjust” command in R.

## Supplementary information

**Additional File 1: Figure S1.** Oligotype distribution of (a) *Propionibacterium*, (b) *Staphylococcus*, and (c) *Corynebacterium* grouped by city and body site.

**Additional File 2: Figure S2.** Differential abundance analysis of taxa significantly associated with city differences within cheek or scalp phenotype (healthy or acne/dandruff). Taxa with DeSeq2 log_2_ fold change > |2| are presented. Taxa in red are fungi, black are bacteria, and green are OTUs within genera known to have biodegradation potentials.

**Additional File 3: Figure S3.** Bacterial and fungal alpha-diversity (a) between cities and sites, as well as between skin phenotype within (b) cheeks (i.e. healthy vs. acne) and (c) scalps (i.e. healthy or dandruff). Statistical significance was tested using the Mann-Whitney test, and FDR-corrected *p*-value <0.05 is considered significant (in bold and underlined).

**Additional File 4: Table S1.** Bacterial and fungal community membership and composition analyses between different sample groups.

**Additional File 5: Figure S4.** Density plots of intra-bacterial (orange), intra-fungal (purple), and inter-domain (green) correlations for (a) healthy and acne-affected cheeks and (b) healthy and dandruff-affected scalps.

**Additional File 6: Table S2**. Significant cross-domain correlations across sample groups based on skin site, city, and skin phenotype.

**Additional File 7: Figure S5.** Association between PAH exposure and microbial diversity. (a) Boxplot depicting position of samples along the first axis of PCA to allocate individuals into groups based on PAH exposure levels as described in Materials and Methods. (b) Histogram showing the number of individuals belonging to one of eight pollutant exposure groups determined as described in Materials and Methods. (c-d) Taxonomic plots showing shifts in overall taxonomic compositions between different PAH exposure level groups for (c) bacteria and (d) fungi. (e-f) Association between pollutant exposure and Shannon diversity for (e-f) bacteria and (g-h) fungi.

**Additional File 8: Table S3.** PAH exposure grouping of individuals.

**Additional File 9: Figure S6.** Sparse canonical correlation analysis (sCCA) of relationships between OTUs and overall PAH exposure levels. (a-b) Heatmaps representing correlations between PAHs and (a) bacterial and (b) fungal OTUs. (c-d) Principal component analysis (PCA) of sample clustering of (c) bacterial and (d) fungal OTUs based on PAH exposure levels. (c) and (d) depict the projection of the subjects in the consensus space.

**Additional File 10: Table S4.** Bacterial and fungal OTUs selected by sparse canonical correlation analysis.

**Additional File 11: Table S5.** Significant relationships between PAH exposure and relative abundance of OTUs, and Sloan model prediction for significant OTUs.

**Additional File 12: Table S6.** Percentage of OTUs fitted within the Sloan model neutral prediction.

**Additional File 13: Table S7.** Taxonomic information of OTUs that deviated from the Sloan neutral model prediction.

**Additional File 14: Figure S7.** Domain-level taxonomic classification of pilot shotgun metagenomics samples. A total of 32 samples were included in the analysis. Taxonomic classification was performed using MetaPhlAn2.

**Additional File 15: Table S8.** Significant associations between city and/or PAH exposure levels and species relative abundance.

**Additional File 16: Table S9.** Significant associations between city, skin site, and/or PAH exposure levels and relative abundance of KEGG orthology genes.

**Additional File 17: Table S10.** Individual and sample metadata including PAH exposure levels.

**Additional File 18: Table S11.** Good's coverage of the bacterial and fungal communities.

**Additional File 19: Table S12.** Metagenomics sequencing quality control information of samples.

**Additional File 20: Figure S8.** Comparison of taxonomic classifications between amplicon sequencing and shotgun metagenomics. Bar plots of the mean relative abundances of the top genera in the 32 samples obtained using amplicon sequencing of the 16S rRNA gene V1-3 region and shotgun metagenomics sequencing.

## Data Availability

Raw amplicon and metagenomics sequences in .fastq format have been deposited in the NCBI short reads archive BioProject PRJNA478488. In-house scripts used for analyses and plotting figures are available at https://github.com/mhyleung/skin_microbiota_pah.
