# Supplementary figures and images for: Changes of the human skin microbiota upon chronic exposure to polycyclic aromatic hydrocarbon pollutants
Source: Microbiome. 2020 Jun 26;8:100. doi: 10.1186/s40168-020-00874-1 (PMC7320578; doi:10.1186/s40168-020-00874-1)

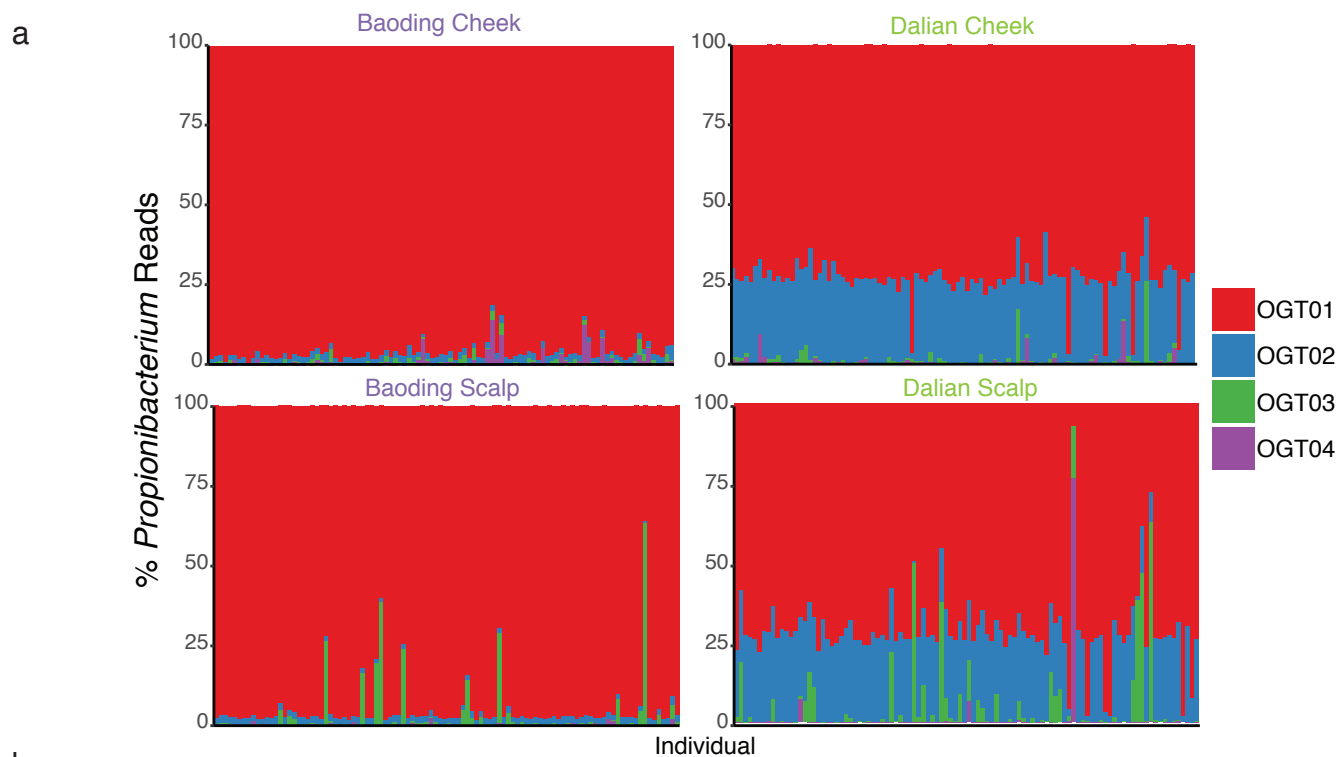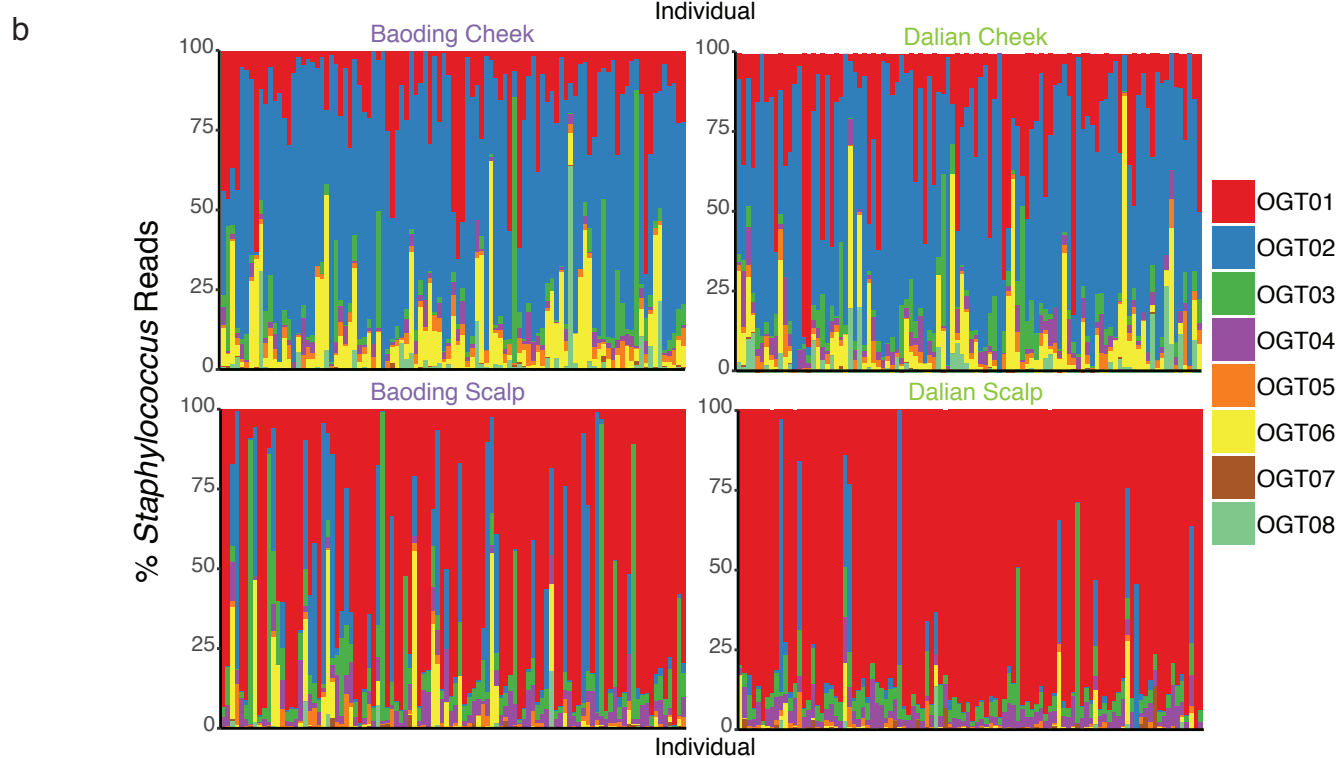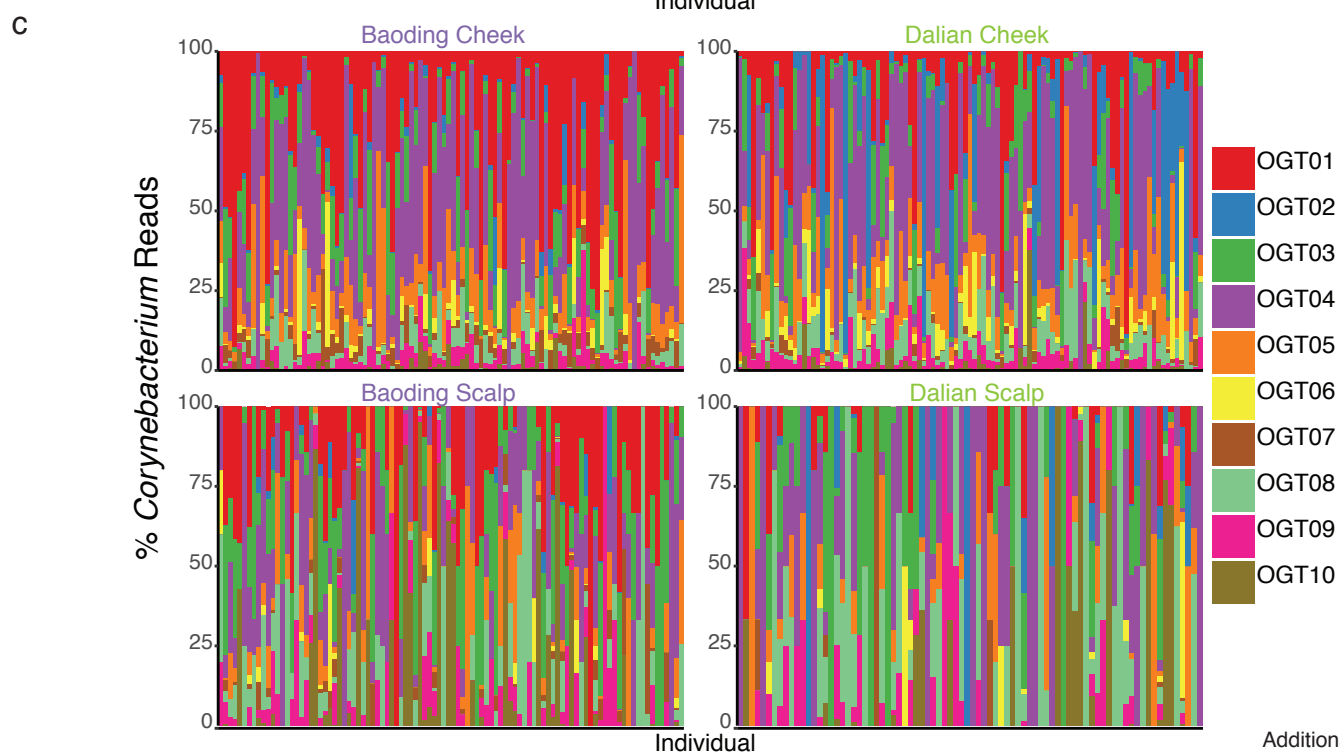

Supplement: Supplementary file 2 — Additional File 1: Figure S1. Oligotype distribution of (a) Propionibacterium, (b) Staphylococcus, and (c) Corynebacterium grouped by city and body site. [file 40168_2020_874_MOESM1_ESM.pdf]

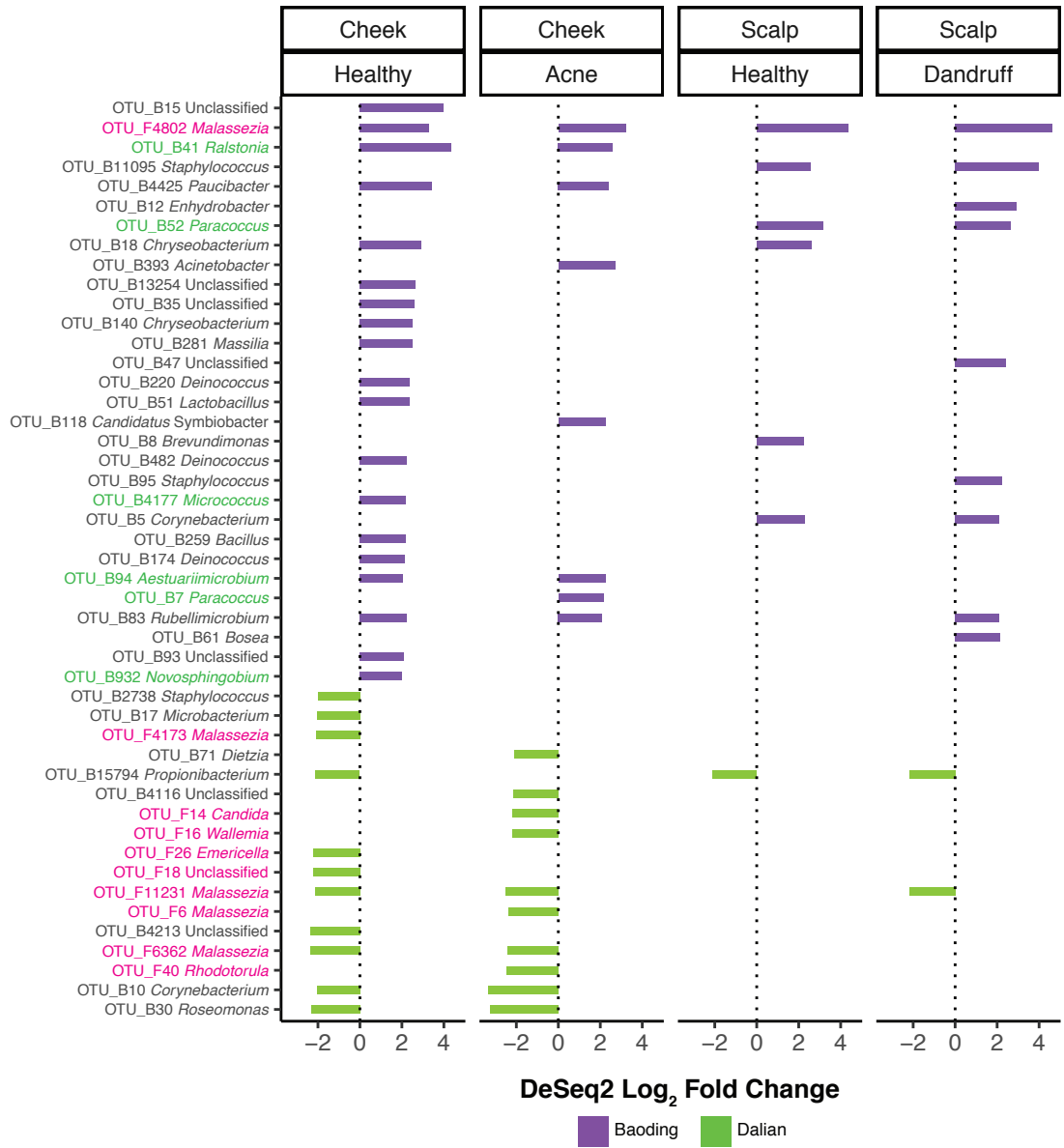

Supplement: Supplementary file 3 — Additional File 2: Figure S2. Differential abundance analysis of taxa significantly associated with city differences within cheek or scalp phenotype (healthy or acne/dandruff). Taxa with DeSeq2 log2 fold change > |2| are presented. Taxa in red are fungi, black are bacteria, and green are OTUs within genera known to have biodegradation potentials. [file 40168_2020_874_MOESM2_ESM.pdf]

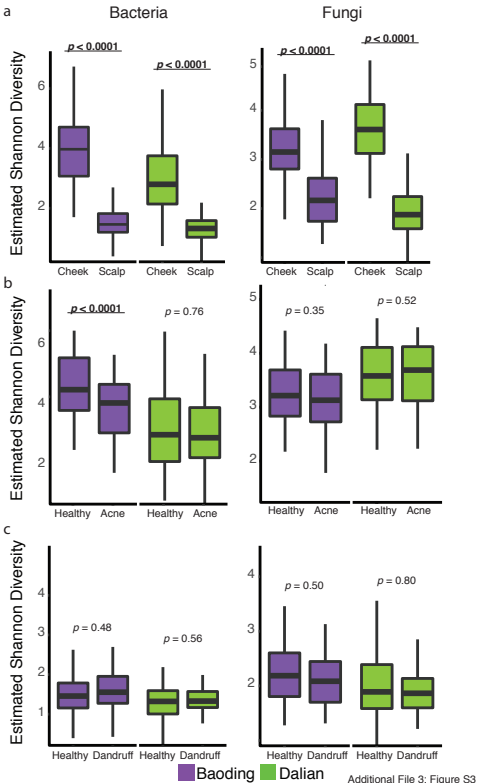

Supplement: Supplementary file 4 — Additional File 3: Figure S3. Bacterial and fungal alpha-diversity (a) between cities and sites, as well as between skin phenotype within (b) cheeks (i.e. healthy vs. acne) and (c) scalps (i.e. healthy or dandruff). Statistical significance was tested using the Mann-Whitney test, and FDR-corrected p-value <0.05 is considered significant (in bold and underlined). [file 40168_2020_874_MOESM3_ESM.pdf]

a

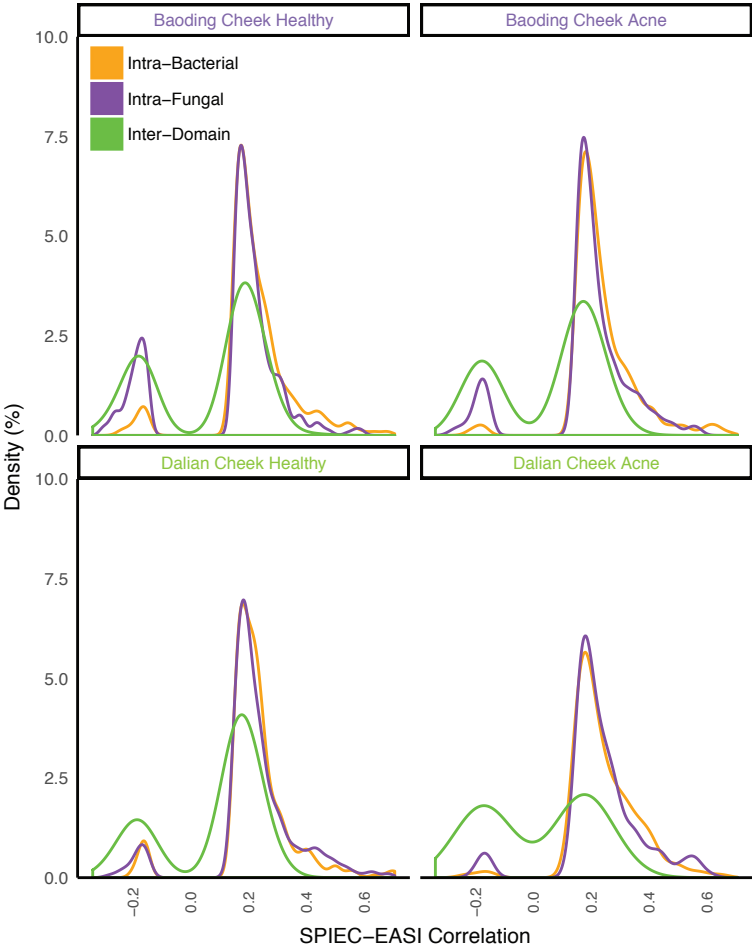

b

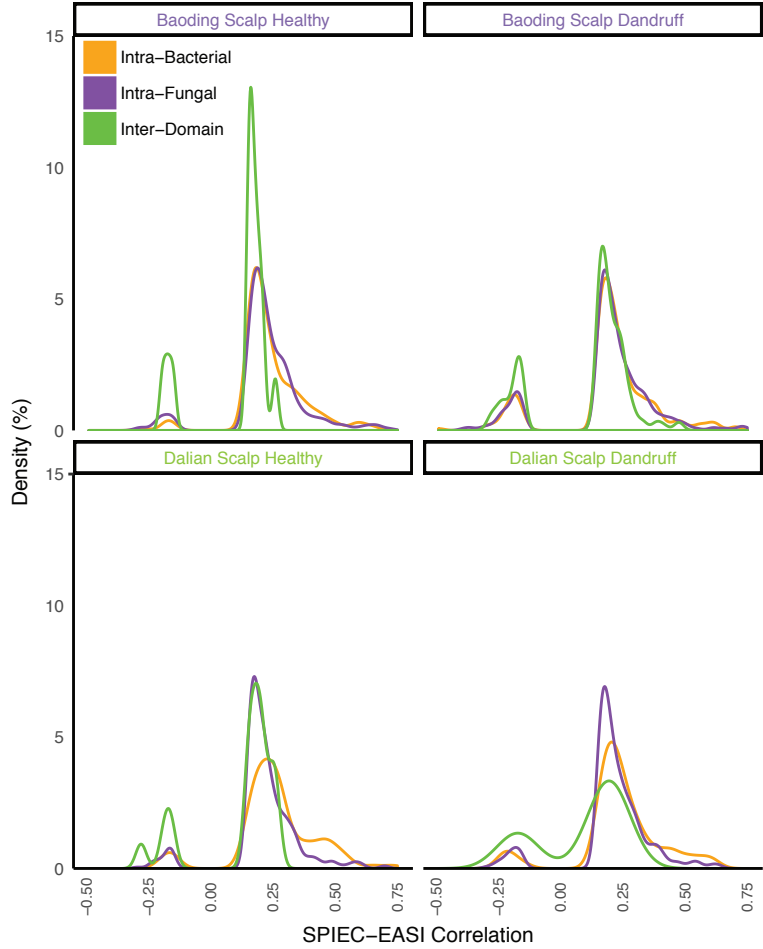

Supplement: Supplementary file 6 — Additional File 5: Figure S4. Density plots of intra-bacterial (orange), intra-fungal (purple), and inter-domain (green) correlations for (a) healthy and acne-affected cheeks and (b) healthy and dandruff-affected scalps. [file 40168_2020_874_MOESM5_ESM.pdf]

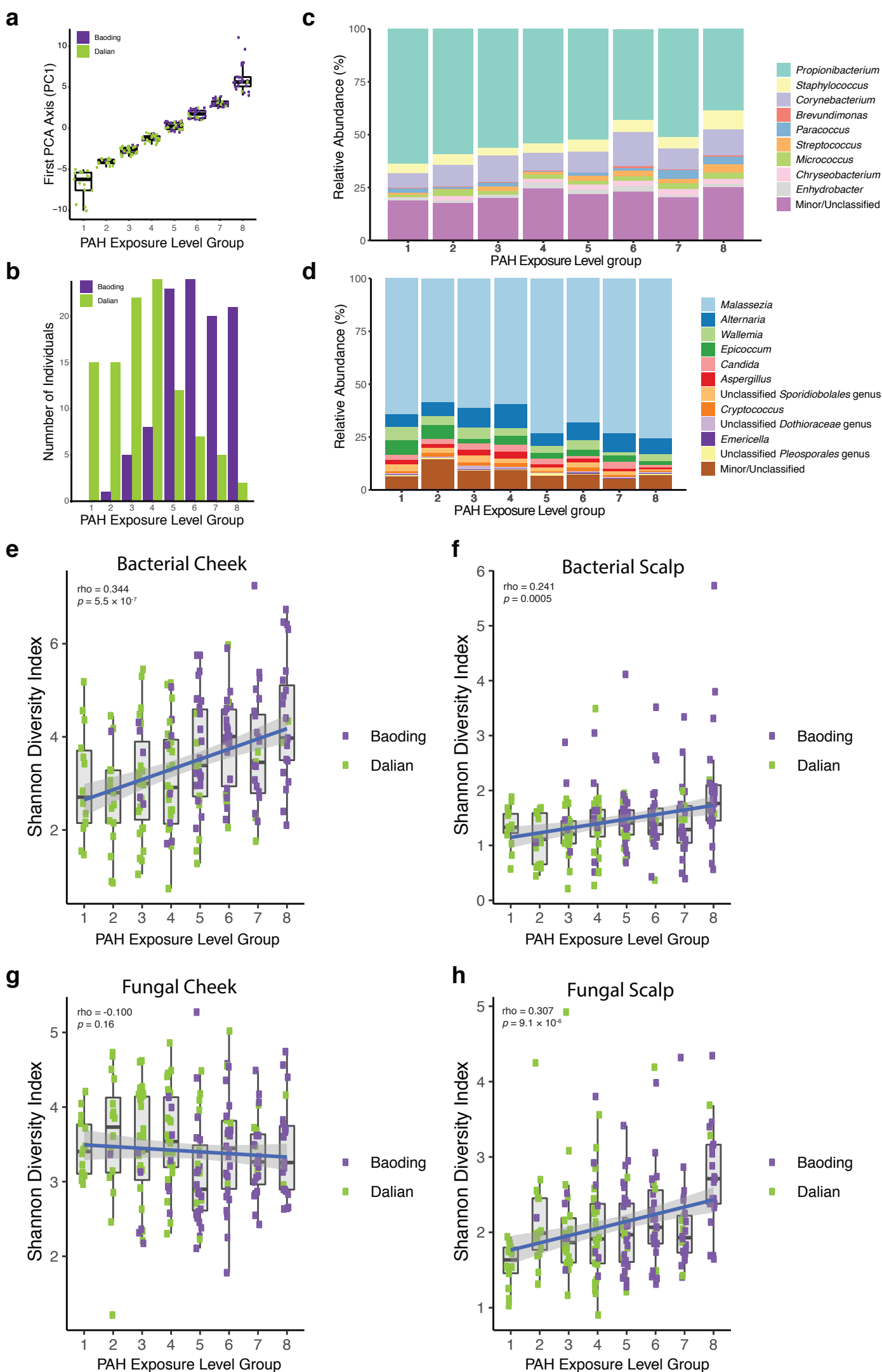

Supplement: Supplementary file 8 — Additional File 7: Figure S5. Association between PAH exposure and microbial diversity. (a) Boxplot depicting position of samples along the first axis of PCA to allocate individuals into groups based on PAH exposure levels as described in Materials and Methods. (b) Histogram showing the number of individuals belonging to one of eight pollutant exposure groups determined as described in Materials and Methods. (c-d) Taxonomic plots showing shifts in overall taxonomic compositions between different PAH exposure level groups for (c) bacteria and (d) fungi. (e-f) Association between pollutant exposure and Shannon diversity for (e-f) bacteria and (g-h) fungi. [file 40168_2020_874_MOESM7_ESM.pdf]

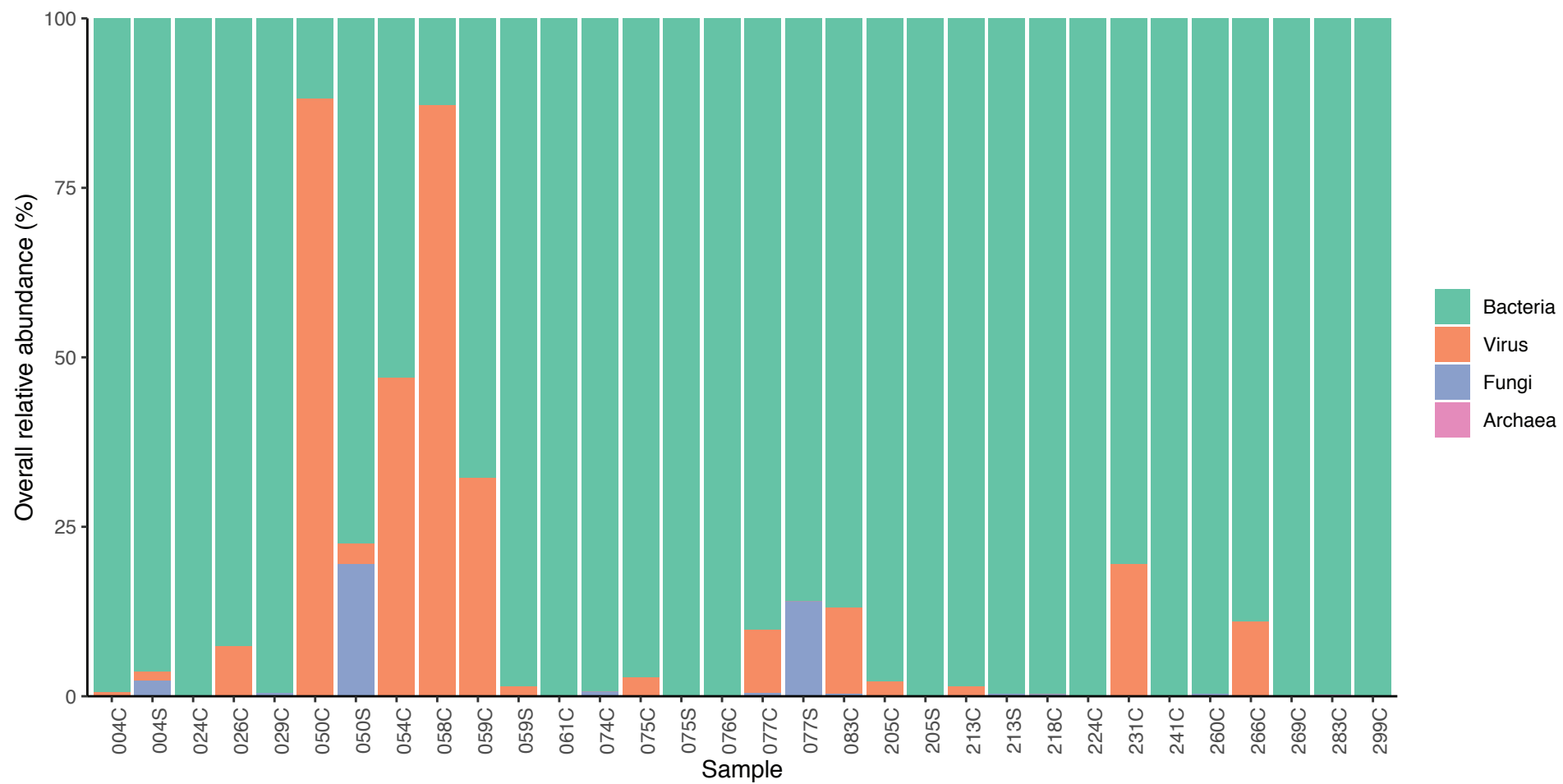

Supplement: Supplementary file 15 — Additional File 14: Figure S7. Domain-level taxonomic classification of pilot shotgun metagenomics samples. A total of 32 samples were included in the analysis. Taxonomic classification was performed using MetaPhlAn2. [file 40168_2020_874_MOESM14_ESM.pdf]

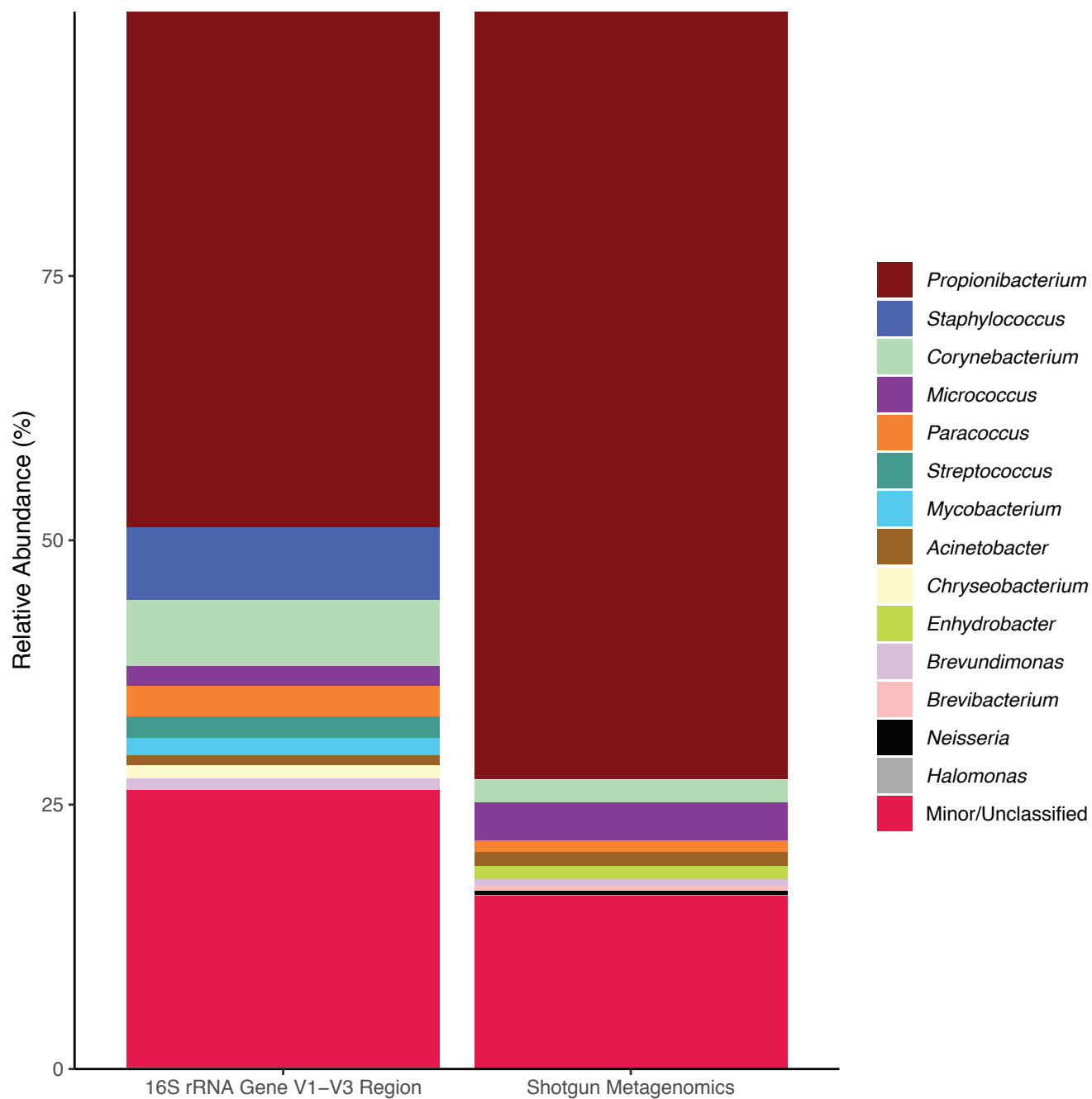

Additional File 20: Figure S8

Supplement: Supplementary file 21 — Additional File 20: Figure S8. Comparison of taxonomic classifications between amplicon sequencing and shotgun metagenomics. Bar plots of the mean relative abundances of the top genera in the 32 samples obtained using amplicon sequencing of the 16S rRNA gene V1-3 region and shotgun metagenomics sequencing. [file 40168_2020_874_MOESM20_ESM.pdf]
