# Supplementary material for: Changes of the human skin microbiota upon chronic exposure to polycyclic aromatic hydrocarbon pollutants
Source: Microbiome. 2020 Jun 26;8:100. doi: 10.1186/s40168-020-00874-1 (PMC7320578; doi:10.1186/s40168-020-00874-1)
Supplement: Supplementary file 10 — Additional File 9: Figure S6. Sparse canonical correlation analysis (sCCA) of relationships between OTUs and overall PAH exposure levels. (a-b) Heatmaps representing correlations between PAHs and (a) bacterial and (b) fungal OTUs. (c-d) Principal component analysis (PCA) of sample clustering of (c) bacterial and (d) fungal OTUs based on PAH exposure levels. (c) and (d) depict the projection of the subjects in the consensus space. [file 40168_2020_874_MOESM9_ESM.pdf]

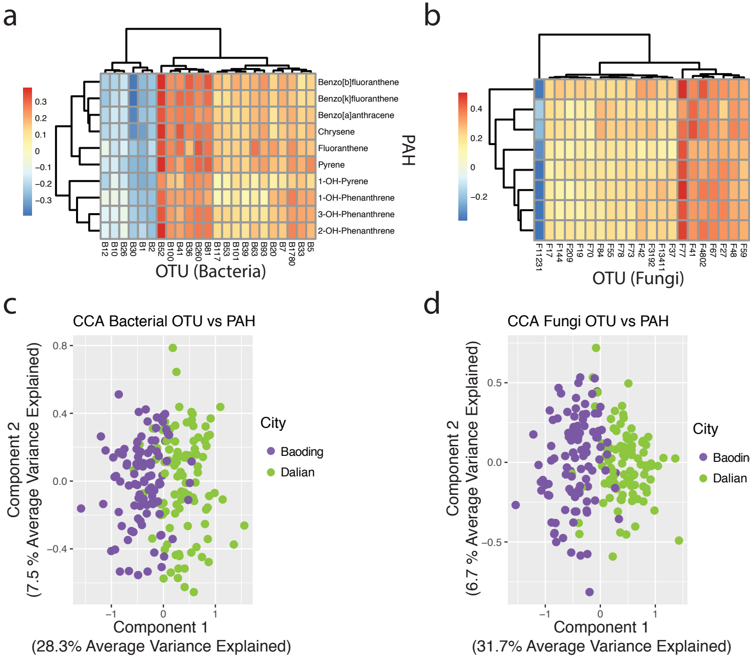

Additional File 9: Figure S6
